# Exploring gender differences in uptake of GP partnership roles: a qualitative mixed-methods study

**DOI:** 10.3399/BJGP.2022.0544

**Published:** 2023-06-27

**Authors:** Laura Jefferson, Su Golder, Holly Essex, Veronica Dale, Karen Bloor

**Affiliations:** Department of Health Sciences, University of York, York.; Department of Health Sciences, University of York, York.; Department of Health Sciences, University of York, York.; Department of Health Sciences, University of York, York.; Department of Health Sciences, University of York, York.

**Keywords:** career choice, career mobility, female, general practice, partnership, physician gender, qualitative research

## Abstract

**Background:**

The unadjusted gender pay gap in general practice is reported to be 33.5%. This reflects partly the differential rate at which women become partners, but evidence exploring gender differences in GPs’ career progression is sparse.

**Aim:**

To explore factors affecting uptake of partnership roles, focusing particularly on gender differences.

**Design and setting:**

Convergent mixed-methods research design using data from UK GPs.

**Method:**

Secondary analysis of qualitative interviews and social media analysis of UK GPs’ Twitter commentaries, which informed the conduct of asynchronous online focus groups. Findings were combined using methodological triangulation.

**Results:**

The sample comprised 40 GP interviews, 232 GPs tweeting about GP partnership roles, and seven focus groups with 50 GPs. Factors at individual, organisational, and national levels influence partnership uptake and career decisions of both men and women GPs. Desire for work–family balance (particularly childcare responsibilities) presented the greatest barrier, for both men and women, as well as workload, responsibility, financial investment, and risk. Greater challenges were, however, reported by women, particularly regarding balancing work–family lives, as well as prohibitive working conditions (including maternity and sickness pay) and discriminatory practices perceived to favour men and full-time GPs.

**Conclusion:**

There are some long-standing gendered barriers that continue to affect the career decisions of women GPs. The relative attractiveness of salaried, locum, or private roles in general practice appears to discourage both men and women from partnerships presently. Promoting positive workplace cultures through strong role models, improved flexibility in roles, and skills training could potentially encourage greater uptake.

## INTRODUCTION

The gender composition of the medical workforce has changed over recent decades around the world. In the UK, women currently make up 53% of the full-time equivalent UK GP workforce.^1^ Despite this near parity in numbers, research has highlighted gender differences in medical working lives (see, for example, Lachish *et al*,^2^ Jefferson *et al*,^3^ and Rodriguez Santana *et al*)^4^ and gendered barriers facing women in medicine.^5^^–^8 Meanwhile, wider societal gender expectations mean that women doctors continue to take on the majority of caring responsibilities in the home,^9^^,^^10^ even in dual-doctor marriages.^11^

A ‘glass ceiling’ has been widely described in medicine, referring to women’s constrained career progression and worse reported pay and conditions.^12^^–^16 A recent independent review^17^ reported that the gender pay gap for GPs was one of the highest of any UK profession, at 33.5% (unadjusted). Although partly explained by women choosing to work fewer hours and differences in age and experience, a substantial pay gap remains. Adjusting to full-time equivalents, women GPs earn 15% less than men.^17^

One factor potentially driving the gender pay gap in general practice is the lower rate at which women become partners — women currently comprise 41% of UK GP partners.^1^ As this more senior position has historically been associated with higher pay and profit-shares, Dacre and Woodmans^17^ estimate that the gap would reduce by 65% if men and women were spread across partnership roles equally.

In this study a rapid literature review (see search strategy in Supplementary Information S1) was conducted, finding sparse recent research evidence exploring barriers to women’s career progression in UK general practice, and no studies focusing directly on gender differences in partnership roles in the past 10 years. Potential reasons for women’s lower uptake of partnerships include higher work pressure,^18^^–^22 spouse’s job location,^23^^–^27 preference for flexibility offered in salaried roles,^18^^,^^28^ and instances of gender discrimination.^29^^–^32 To inform policy around partnerships and the gender pay gap this mixed-methods study was conducted (reported in full at york.ac.uk/prepare-reports).

## METHOD

A mixed-methods research design was employed, including a secondary analysis of existing qualitative interviews, social media analysis of UK GPs’ Twitter commentaries, and asynchronous online focus groups with GPs in England. The findings were combined using methodological triangulation.

### Secondary analysis of qualitative interviews

Existing transcripts of 40 interviews were analysed from the authors’ recent study exploring the impact of COVID-19 on GP wellbeing,^33^ which included discussion of barriers relating to partnership and careers. A detailed summary of the interview methods are provided in Jefferson *et al*.^33^ NVivo 12 was used, iteratively coding data including the terms ‘salaried’ and ‘partner.’ This provided useful contextual information and helped to inform the conduct and design of the focus groups.

**Table:** How this fits in

An unadjusted gender pay gap of 33.5% exists in general practice, reflecting partly the differential uptake of partnerships among women GPs. This study used a mixed-methods approach to explore factors affecting uptake of partnership roles, focusing particularly on gender differences. Factors at individual, organisational, and national levels influence partnership uptake and career decisions of both men and women GPs. Gender differences were apparent with women reporting greater challenges balancing work–family, negative working conditions, including maternity and sickness pay issues, and discriminatory practices perceived to favour men and full-time GPs. Promoting positive workplace cultures through strong role models, improved flexibility in roles, and skills training could potentially encourage greater uptake among both men and women.

### Social media analysis

Practising UK NHS GPs on Twitter (see Golder *et al*^34^ for details) were identified using user descriptions (‘GP’, ‘G.P.’, ‘general practitioner’, ‘General Practitioner’, ‘MD’) via Mozdeh (big data text analysis software http://mozdeh.wlv.ac.uk/). This located 1924 UK NHS GPs (after restricting by UK location and NHS practice), from which 1 213 202 of the most recent tweets (maximum of 3200 tweets per user) were downloaded. These tweets were then searched for the following terms:

- GP AND Partner (1067 tweets);
- GP AND Partnership (451 tweets);
- GP AND Principal (38 tweets);
- Partner AND Locum (137 tweets); and
- Salaried (1565 tweets).

Gender was automatically extracted using Mozdeh software based on first names as male, female, or unclassified. For unclassified users in the current study the authors relied on self-identification (use of pronouns such as ‘she/her’, descriptions such as ‘busy mum’, ‘wife of …’, or a combination of name and self-photo[s]). Where this was not possible, gender was categorised as ‘unknown’. GPs’ city or town was extracted, although some only provided the location as ‘UK’.

After removing duplicates, 1886 tweets posted from 1 January 2019 to 12 March 2022 were analysed. Each tweet was screened for relevance to the study aims, with 1257 tweets excluded at this point. The remaining tweets were coded using a framework that emerged inductively. Although Twitter data are in the public domain, to preserve the anonymity of the GPs, tweets are paraphrased when presented in the current study’s results.

### Asynchronous online focus groups

Asynchronous online focus groups (AOFGs) provide the opportunity to explore the views of groups of geographically dispersed individuals remotely, connecting on a topic at a time of their choosing.^35^ This can be particularly beneficial for discussions with hard-to-reach groups, including medical professionals.^36^ AOFGs generally take place over the course of several days via online discussion boards, facilitated either through specific platforms or social media groups.

An online focus group platform (Collabito) was used, accessible through a computer or mobile phone and enabling participants to contribute at multiple time points at their convenience. Topics were introduced daily over 5 days to explore GPs’ views of taking on partnership roles and relevant experiences during their working lives. Initial questions were non-leading, framed openly, and facilitators prompted with follow-up questions if debate stalled. Discussions were monitored for appropriate content and member checking was conducted when any comments were unclear.

### Sampling and recruitment

The sampling framework captured experiences of different groups of GPs, varying in role type and gender, but weighted towards women:

- partners (two groups of women, one group of men);
- non-partners (two groups of women, one group of men); and
- those who had left partnership roles (mixed gender group).

The target sample was 8–10 participants in each focus group (56–70 in total). Participants were recruited through snowballing local and national networks of contacts, social media promotion (primarily Twitter), and email circulation within key organisations. A goodwill voucher payment of £75 was provided to each participant.

One focus group (with salaried women GPs) was conducted as a pilot to test the processes before wider dissemination. Data for this pilot group were comparable with the wider discussions and were therefore used in analysis.

### Analysis

Transcripts from each AOFG session were entered into data-sorting software, NVivo 12. The process of framework analysis^37^ was used to analyse the data thematically, moving through the stages of data familiarisation, sorting the data into emerging themes and exploring relationships between themes. The findings were contextualised using quotations throughout. One researcher undertook coding, with wider team consultation when developing and refining the coding framework.

Gender differences were primarily explored qualitatively but, owing to the large number of comments in the datasets, it was possible to conduct statistical significance testing. A test of proportions was used, with significance set at *P* = 0.05, to explore whether there were statistically significant differences in the proportion of men and women GPs commenting on certain themes.

### Data integration

Each dataset was analysed separately in the first instance, then they were combined iteratively using the principles of convergent mixed-methods designs.^38^ The social media analysis provided broad insights relating to factors that have an impact on GP partnership uptake among a large, reasonably generalisable group. Focus groups and interview findings provided more detailed understanding of GPs’ experiences. As all forms of data were essentially qualitative and there was good fit across the datasets, the findings are presented combined.

### Reflexivity

A reflexive approach was maintained throughout the design and analysis stages to limit potential for pre-conceptions to influence research findings. All researchers were female, with non-medical backgrounds; to improve credibility and reliability the findings were discussed with GP stakeholders, and researcher triangulation throughout data collection and analysis was undertaken.

## RESULTS

### Participant characteristics

The data were derived from 40 interviews with UK GPs during spring/summer 2021, 232 GPs tweeting relating to GP partnership roles from January 2019 to March 2022, and 50 GPs participating in AOFGs in May 2022. Reflecting the study aims, interview and focus group samples included a higher proportion of women GPs (interviews 29 women and 11 men, AOFGs 36 women and 14 men). Variation in career stage, job roles, and location were achieved, although there was a higher proportion of GPs from the Yorkshire region (see Tables 1 and 2). The social media dataset comprised 347 (55%) tweets by 135 men, 269 (43%) tweets by 92 women, and 13 (2%) tweets posted by 5 GPs of unknown gender.

**Table 1.** Interview participant characteristics

**Characteristic**	**Value (*n* = 40)**
**Career stage, *n* (%)**	
Early	13 (33)
Established	19 (48)
Late	8 (20)

**Gender, *n* (%)**	
Male	11 (28)
Female	29 (73)

**Age, years, *n* (%)**	
<30	3 (8)
30–39	20 (50)
40–49	9 (23)
50–59	6 (15)
>60	2 (5)

**Ethnicity, *n* (%)**	
Black, Asian, or other ethnic minority	10 (25)
White British	27 (68)
White non-British	3 (8)

**Role, *n* (%)**	
GP trainee	6 (15)
GP retainer	1 (3)
Salaried GP	17 (43)
GP partner	14 (35)
Retired GP	2 (5)

**Location, *n* (%)**	
East of England	3 (8)
London	5 (13)
North East	1 (3)
North West	3 (8)
South East	3 (8)
South West	4 (10)
West Midlands	5 (13)
Yorkshire and Humber	14 (35)
Northern Ireland	2 (5)

**Clinical sessions, median (IQR)**	6 (4.0‒1.6)

**Clinical sessions, *n* (%)**	
1–4	11 (28)
5–7	16 (40)
≥8	9 (23)
Retired	2 (5)
Unknown	2 (5)

**Portfolio roles, *n* (%)**	18 (45)

**Area demographics, *n* (%)**	
Highly deprived	10 (25)
Pockets of deprivation	9 (23)
Rural or semi-rural	4 (10)
Large population of older adults	4 (10)
Missing	13 (33)

*IQR = interquartile range.*

**Table 2.** Focus group participant characteristics^a^

**Characteristic**	**All (*n* = 50)**	**Women salaried group 1 (*n* = 7)**	**Women salaried group 2 (*n* = 8)**	**Women partners group 1 (*n* = 8)**	**Women partners group 2 (*n* = 7)**	**Men salaried (*n* = 7)**	**Men partners (*n* = 6)**	**Former partners (*n* = 7)**
**Gender, *n* (%)**								
Male	14 (28)	—	—	—	—	—	—	1
Female	36 (72)	—	—	—	—	—	—	6

**Age, years, *n* (%)**								
<30	1 (2)	0	1	0	0	0	0	0
30–45	32 (64)	6	5	3	3	7	4	3
46–60	14 (28)	1	2	4	4	0	2	1
>60	3 (6)	0	0	0	0	0	0	3
Missing	0	0	0	1	0	0	0	0

**Working hours, median (IQR)**	13 (19)	25.0 (12.5)	22.5 (9.4)	40.0 (14.5)	33.0 (6.0)	35.0 (5.8)	40.0 (1.9)	18.0 (9.4)

**Caring responsibilities, *n* (%)**								
No caring role	17 (43)	1	3	0	3	3	3	3
Caring, dependent children	31 (62)	6	5	7	4	4	3	3
Caring, other	1 (2)	0	0	1	0	0	0	0
Missing	1 (2)	0	0	0	0	0	0	1

**Practice size, *n* (%)**								
<10 000	8 (16)	0	1	2	0	4	0	1
10 000–15 000	12 (24)	1	2	1	1	2	1	3
15 001–20 000	9 (18)	0	2	3	2	0	2	1
>20 000	18 (36)	6	2	2	4	1	3	1
Missing	3 (6)	0	1	0	0	0	0	1

**Role, *n* (%)**								
Locum	4 (8)	—	—	—	—	—	—	—
Salaried	24 (48)	—	—	—	—	—	—	—
Partner	21 (42)	—	—	—	—	—	—	—
Retired partner	1 (2)	—	—	—	—	—	—	—

**Location, *n* (%)**								
East of England	4 (8)	—	—	—	—	—	—	—
London	4 (8)	—	—	—	—	—	—	—
North East	1 (2)	—	—	—	—	—	—	—
North West	4 (8)	—	—	—	—	—	—	—
South East	5 (10)	—	—	—	—	—	—	—
South West	3 (6)	—	—	—	—	—	—	—
West Midlands	6 (12)	—	—	—	—	—	—	—
Yorkshire & Humber	23 (46)	—	—	—	—	—	—	—

a *Location and role data not provided by focus group to preserve participant anonymity. IQR = interquartile range.*

### Thematic findings

**Figure 1.**
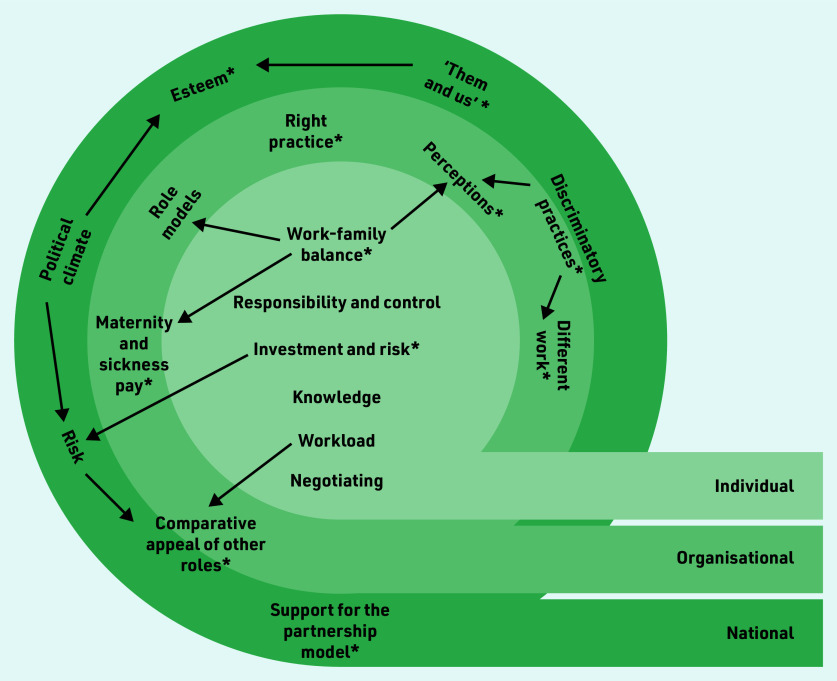
*Factors influencing perceptions and experiences of uptake of partnership roles. Relationships between themes are indicated by arrows and themes with gender differences in views or experiences are highlighted with asterisks.*

The inclusion of both men and women GPs in the datasets not only allowed a comparison across gender, but also highlighted how many facilitators and barriers were relevant across genders. The themes were organised into ‘individual,’ ‘organisational’, and ‘national’ levels (illustrated in Figure 1). Relationships between themes are indicated by arrows, which show how barriers often crossed levels, for example, the impact of childcare needs and the desire to achieve work–family balance was influenced by organisational factors, such as role models, maternity pay, and presumptions of managers or practice teams. Themes with gender differences in views or experiences are highlighted with asterisks. Themes, with frequency of coding and gender breakdown, are provided in Tables 3 and 4.

**Table 3.** Frequency of coding, number, and proportion of women and men discussing each theme

**Focus group themes and sub-themes**	**Women and men discussing each theme**			

	Women (*n* = 36)		Men (*n* = 14)	

	*n*	%	*n*	%
**Times change**	17	47	6	43

**Barriers**				
Work–family life^a^^,^^b^	31	86	8	57
Financial	31	86	12	86
Workload	26	72	10	71
Risk	21	58	8	57
Relationships — right practice^a^	19	53	10	71
Business management	28	78	10	71
Knowledge	25	69	8	57
Media and political narrative	13	36	5	36
Other ways to earn/changes^a^	11	31	7	50
Perceptions/awareness	7	19	2	14
Role models	3	8	1	7
Culture — us and them^a^	15	42	2	14
Other	3	8	2	14

**Facilitators**				
Responsibility and control	29	81	11	79
Relationships^a^	19	53	5	36
Investment^a^	13	36	9	64
Knowledge	7	19	1	7
Esteem^a^	7	19	5	36
Flexibility	3	8	1	7
Other	7	19	3	21

**Gendered**				
Barriers	32	89	11	79
Family/childcare	26	72	11	79
Discrimination^a^^,^^b^	20	56	3	21
Imposter syndrome^a^	7	19	1	7
Different work^a^	5	14	0	0
Career choice^a^	7	19	1	7
Negotiating^a^	9	25	2	14
Maternity	15	42	5	36
Gender pay gap causes	8	22	4	29
Perceived parity^a^^,^^c^	14	39	10	71
Cultural	1	3	2	14

**New to partnership**				
Positives	23	64	8	57
Negatives	29	81	12	86

a *Indicates larger differences in proportion of responses by gender.*

b *Statistically significant difference,* P *= 0.03.*

c *Statistically significant difference,* P *= 0.04.*

**Table 4.** Topics covered by tweets, by gender^a^

**Social media theme**	**Total, *N* = 629 tweets**		**Male, *N* = 375 tweets**		**Female, *N* = 291 tweets**	
	***n***	**%**	***n***	**%**	***n***	**%**
Barriers to partnership roles	202	32	120	35	78	29
No aspirations to be partner	37	6	19	6	18	7
Facilitators to salaried roles	19	3	11	3	7	3
Facilitators to locum roles	11	2	5	1	6	2
Opposed to partnership model	58	9	33	10	25	9
Facilitators to partnership roles	31	5	15	4	16	6
Aspirations to be a partner	15	2	6	2	9	3
Barriers to salaried roles	86	14	45	13	39	15
Barriers to locum roles	11	2	7	2	3	1
Support for the partnership model^b^^,^^c^	89	14	58	17	27	10
Childcare, conditions, and pay^b^^,^^c^	32	5	11	3	21	8
Mix of GPs	42	7	21	6	19	7
Other (pathways, training …)	47	8	24	7	23	9

a *Percentages are given as proportion of total tweets (*N*), with breakdown by gender. Unknown gender tweets were not thematically coded (*n *= 5).*

b *Indicates larger differences in proportion of responses by gender.*

c *Statistically significant difference* P *= 0.01.*

Many of these factors are interrelated and the comparative attractiveness of partner roles was counteracted by the appeal of salaried, locum, or private provider roles, which were described as offering greater flexibility and, in some locations, similar pay.

### General changes over time

All the datasets reflected changes over time, including societal changes, changes in medical culture, perceptions of and appetite for partnership roles, and the processes of buying-in. Societal changes and changes to medical training such as the provision of ‘less than full time’ training routes were thought to make medicine more accessible to women. Wider changes seen as deterring GPs (both men and women) from taking on partnership roles included higher costs of student loans, personal mortgages, perceived risk including instability in UK general practice, and unmanageable workload pressures.

### Individual factors

#### Work–family balance

Childcare responsibilities were described (by both men and women) as the greatest barrier to women’s career progression as they attempt to balance work–family lives. Although also presenting challenges for men, women were more likely to describe making career decisions to facilitate family life. High childcare costs discouraged women from increasing hours, although others described a greater ability to cope with workload demands when working part-time. Cultural expectations were mentioned, with participants from Asian backgrounds describing the greater pressure of traditionally women’s roles in the home and traditionally men’s role in providing financial security.

Salaried roles enable greater flexibility, but some women also felt reluctant to join partnerships as their children grew up. Particularly true in dual-doctor marriages, men’s faster progression to more senior roles while women take maternity leave and work part-time were described as leading to their lower comparative earnings and less financial incentive, and ability, to join partnerships:

*‘It feels as though we may spend our twenties training, thirties having babies, forties doubting ourselves and feeling our male counterparts have already taken all the leadership roles, and fifties wanting to retire … partnership needs to become more flexible and understanding of the needs and skills women in their 30s offer in order to be more attainable.’* (Female salaried GP, 30‒45 years, focus groups)

*‘My wife is also a GP and has taken maternity leaves meaning my career progressed and I became a partner before she had the chance or desire to do it. Then, from a purely financial decision it makes more sense for me to work more than her.’* (Male GP partner, 30‒45 years, focus groups)

#### Workload and responsibility

The responsibility and ability to shape local services was the greatest motivator for partnership uptake, for both men and women:

*‘The appeal of GP partnership is that it grants us the freedom to influence our job, which can be thrilling or frightening but is ultimately a privilege. It is empowering.’* (Male GP partner, tweet, paraphrased)

Many GPs, however, felt the responsibility for business management and accountability for wider roles (such as human resources [HR], finances) was too onerous alongside clinical workload pressures. This applied to both men and women, particularly younger salaried GPs who were dissuaded from partnerships by seeing stress and burnout among partner colleagues. One described that a:

*‘hidden curriculum of “you just do whatever is needed in time that doesn’t exist” no longer works.’* (Female salaried GP, 30‒45 years, focus groups)

Younger GPs appeared to be less willing to accept these conditions:

*‘A partner must work 70-hour weeks and perform even more non-clinical administrative duties than a salaried GP. It is gruelling. Many young general practitioners view salaried and locum work as allowing them to do what they were trained in — consulting with and assisting patients rather than battling targets and non-clinical duties.’* (Female GP partner, tweet, paraphrased)

Women GP partners in this sample were more likely to work fewer sessions and described doing so to balance their working and home lives. They described partnership as: *‘not a 9–5 job, more a 5–9 job’* (female GP partner, focus groups), with general practice running on the goodwill of partners working beyond contracted hours. Both men and women commented on this being more prohibitive for women, because of historically gendered roles in the home. It may also be possible that women partners experience different patterns of work in practices — some women described being given a larger share of children’s or women’s health appointments or responsibility for supporting teams — which were described as leading to increased workloads.

Stress and burnout caused some GP partners to leave partnership roles: issues that had been exacerbated over recent years. Those remaining described extreme pressure because of having no incumbents to replace them, and feeling concerned about their commitment to local communities.

#### Investment and financial risk

The cost of buy-in was viewed as prohibitive by many (both men and women), who likened this to a ‘second mortgage’ and found this unachievable because of family circumstances, personal mortgages, or university debts. The perceived uncertainty around the future of the partnership model influenced GPs’ willingness to take this on, as did wider economic concerns. Some GP partners described the move to partnership through gradual buy-in schemes as initially decreasing their earnings. Variability of GP partner income was also viewed as problematic, as were staffing fluctuations, partners leaving, employee pay rises, pension changes, and indemnity insurance costs:

*‘It makes me nervous when partners leave and no new ones join as our capital share has to increase. I worry about this money more than I do about annual drawings and profit share as I see it as money which could potentially be lost and never returned at the point of retirement. It can feel like a bottomless pot.’* (Female GP partner, 30‒45 years, focus groups)

*‘Under the current model partners are only able to increase GP capacity by taking a considerable pay cut. Why would my partners and I consent to accepting a huge pay reduction when we have never worked so hard?’* (Male GP partner, tweet, paraphrased)

Gender differences were observed in terms of differential financial motivation (both short-term in the form of pay and longer-term in relation to investment in buildings etc.). Men were more motivated by financial benefits (9/14 [64%] men versus 13/36 [36%] women reported feeling motivated by this):

*‘I am glad I joined the partnership as financially it has been very wise. My pay essentially doubled.’* (Male GP partner, 46–60 years, focus groups)

Partners (both men and women) generally felt that the financial rewards of partnership, coupled with ability to shape practices, counteracted their greater stress and workload:

*‘It is stressful but I get paid more than four times the average UK salary for doing three days a week.’* (Male GP partner, 46‒60 years, focus groups)

#### Knowledge

Insufficient training to prepare for partnerships was reported by many participants, and they apparently knew little of the ‘new to partnership’ scheme, which aims to encourage uptake. In total 14 of 50 focus group participants were unaware of the scheme and 33 of 50 wanted more information to support their decision. Further training in the business skills required for partnership was cited, either during medical school or beyond, although some were uninterested in this element of the role:

*‘I feel turned off by and unsuited to the business aspect of partnership, thus was never attracted to it, despite the excellent pay raise. We aren’t suitably trained in medical school for partnership roles and I don’t believe all general practitioners and doctors are naturally good business people.’* (Female salaried GP, tweet, paraphrased)

### Local and organisational factors

#### Discriminatory practices

Women GPs reported that their working lives and career decisions were influenced by gendered behaviours including overt sexism, differential treatment by colleagues and patients, lower respect, and gendered societal norms:

*‘Medicine still feels very patriarchal, valuing working long hours, devaluing part-time workers. I have suffered micro-aggressions from patients and partners. An ex-partner completely changed his attitude towards me once I had kids and went part-time.’* (Female GP partner, 46–60 years, focus groups)

Prejudices about women’s roles in the home affected some conversations with senior colleagues about partnership roles:

*‘I have spoken to partners at my current practice who have suggested I might not be ready for partnership as a new mum, the responsibilities may be too much and I might be better to wait. For me being a new mum doesn’t put me off partnership.’* (Female salaried GP, 30–45 years, focus groups)

When asked about potential causes of the gender pay gap in general practice, around half of the focus group sample commented on parity in pay among GPs in their practices. There was a tendency for male GPs to attribute the gap to differences in working hours or uptake of partnership roles, whereas women were more inclined to cite women’s greater reluctance to negotiate pay and feelings of ‘imposter syndrome’. Women feared potential escalations, describing peers’ experiences of legal proceedings with employers because of disagreements on pay and the impact this had on working relationships:

*‘I have a colleague who feels indebted to her surgery due to flexibility of childcare and recent maternity leave. She almost feels greedy asking for a raise.’* (Male salaried GP, 30–45 years, focus groups)

#### Maternity and sickness pay

Women described greater perceived financial security in salaried roles because of different contractual conditions around maternity and sickness pay, which can vary practice-to-practice according to partnership arrangements. Women had a lack of awareness of policies for maternity pay within their practice, often presuming these would be more prohibitive for partners than salaried GPs. Women described this as influencing their decisions around partnership uptake and some experienced the financial impact from paying locums during maternity leave:

*‘Whereas most salaried positions follow a standard BMA* [British Medical Association] *contract, depending on the partnership there is every chance I could have to pay for the locum to cover me while I am off — which is difficult to find and expensive.’* (Female salaried GP, 30–45 years, focus groups)

This may be particularly challenging if occurring during the initial period following buying into a practice, because of high repayments of business loans or gradual buy-in arrangements having an impact on pay:

*‘I had to pay my maternity locum out of savings as my income from the practice* [was] *much lower than hers as it took me three years to reach full parity* [following a gradual buy-in process]*.’* (Female GP partner, 46–60 years, focus groups)

#### Team working

Practice dynamics were important, in particular supportive teams, with women partners often describing how they had been mentored by positive role models:

*‘*[Partnership can be] *very supportive and very rewarding but the key is to be lucky with your partners. Choosing the right team to be part of is more important than any other factor.’* (Female GP partner, tweet, paraphrased)

Those who struggled with or had left partnerships often reported challenging working relationships (more common in men than women). Both genders voiced concerns about smaller practices and risk of being the ‘last man standing’.

Women GPs from larger practices described feeling that their voices were not heard, resulting in disengagement. Some had broached more flexible working hours, even in larger teams, but experienced ingrained cultures and an unwillingness to change. Conversely, larger organisations were described as benefiting from business and HR managers that reduced the responsibilities of partners.

### National factors

#### Risk and political climate

Views of partnership were influenced, across both genders, by the wider political context and pressures facing general practice. GPs voiced concerns about potential changes to the organisation of general practice, the future of the partnership model, and *‘dwindling partner numbers’* (Female partner, 46–60 years, focus groups), particularly in underserved areas:

*‘My biggest worry about joining a partnership at the moment is the partnership model may not exist in ten years.’* (Female salaried GP, 30–45 years, focus groups)

GPs felt pressure from public expectations to provide a *‘*[cradle] *to grave care with demands of Amazon Prime instant delivery of service.’* (Interview participant, male GP partner, 30–45 years).

GPs described feeling undervalued by the government and general public as views of the profession had shifted and there was an increasing sense of reluctance to accept personal financial risk (described earlier) amid these changes:

*‘We are the only NHS doctor group who would commit to such levels of personal borrowing to provide clinical services. We accept high financial risk … as the partnership model becomes more under threat and public opinion shifts − I think I might struggle to maintain this level of comfort with such financial risk.’* (Male GP partner, 30–45 years, focus groups)

#### Esteem

Conversely, some GPs described a sense of pride in the contributions they made and their identity was closely linked to their esteem:

*‘I always saw myself as a partner … I thought being a partner was the ultimate in GP* [general practice]*.’* (Male partner GP, 46‒60 years, focus groups)

These comments were most commonly made by men and some older women, who cited ambitions and an expectation to move into these roles. There was a sense that aspirations were changing over time, and this was affected by recent negative media portrayals.

#### Role demarcations: ‘Them and us’

A ‘them and us’ culture emerged in the data, with demarcations arising between salaried and partner GPs. This was more commonly described by women; whereas some GP partners voiced frustrations with salaried GPs’ lower workload and more defined working hours, salaried GPs expressed frustration and powerlessness, and described feeling as if work was ‘farmed out’ to them.

## DISCUSSION

### Summary

This combined analysis across a large body of qualitative data highlights individual, organisational, and national factors that may prevent GPs taking on partnership roles, and particular gendered experiences that may inhibit women’s uptake.

Key barriers included workload, greater responsibility, financial investment, and risk. Partnership was viewed as unappealing amid wider workforce issues, potential changes to the partnership model, and perceived lowered esteem of UK general practice. Some viewed partnerships as less attractive relative to other roles, particularly when earning potential is similar (this appears to vary with location) and there is believed to be greater opportunity for work–life balance in salaried and other roles. Some conflict was described between these groups of GPs, with a ‘them and us’ culture emerging.

These challenges appear to affect both men and women, although there were gender differences in perceptions and experiences including women’s more negative experiences of childcare pressures and balancing work and family life, working conditions including maternity and sickness pay, and discriminatory practices. Men GPs on social media were more favourable towards the partnership model.

The importance of ‘finding the right practice’ was stressed, with examples of this working well to support GPs to join partnerships, share workloads, and provide a sense of teamwork. Women GP partners described valuing positive role models, having open conversations about partnership, and the positive influence of working for a shared goal. Patriarchal practices were reported: some participants felt that the work of women and part-time workers was devalued and many women described experiencing toxic behaviours.

### Strengths and limitations

Pooling data across 40 interview participants, 50 focus group participants and 629 social media tweets (relating directly to partnership roles) by 232 GPs created a large body of qualitative data on which to base the analysis. This mixed-methods design with triangulation across three methods strengthens the validity and transferability of the findings.

Although participants were recruited through a variety of channels for the online focus groups, the majority of participants came through snowball sampling of existing contacts and Twitter, which was an efficient method for recruiting GPs but can result in reduced representativeness of views, for example, 46% of the focus group sample came from the Yorkshire and Humber region. The authors also had reason to believe that a fraudulent individual entered two of the focus groups, impersonating a doctor to receive the gift voucher for participation. This became apparent during conduct of the focus groups and when checking each individual’s General Medical Council number to issue vouchers. As a result, this participant’s comments were excluded from analysis and the sample. There is no reason to think that the focus group discussions were affected adversely by this incident; discussion among the remainder of these groups was lively and relevant.

Using social media for this study provided additional contextual information from a wider dataset than traditional qualitative methods, but this also comes with challenges owing to how gender is determined. In this study primarily two methods were relied on: gender determined by the Mozdeh social media software package and self-identification (for example, using ‘she/her’ pronouns or descriptions). Self-identification may be preferable for future research given the challenges of assigning participants to gender groups. Nevertheless, level of error is likely to be small and in this study the similarities in findings across other methods suggests this has not had an impact on this study.

### Comparison with existing literature

It has been over 20 years since Reed and Buddeberg-Fischer^5^ summarised the struggles affecting women doctors’ career progression, and there has been limited research since then. The findings in the current study highlight the continued challenges around inflexibility, balancing work–family lives, and discriminatory practices. A recent international study of female doctors’ career progression in primary care reported similar findings.^39^ Many existing studies in this field report women’s views without comparing men’s experiences. In the current study, although gender differences were apparent in some areas, other barriers were felt by both men and women.

### Implications for research and practice

Concerns around the security of investment in partnerships, amid recurring debates about changing the partnership model, along with the negative impact of media portrayals on GP esteem, highlight a need for a change in the narrative around general practice as a profession.

Greater standardisation of partnership contracts could improve perceptions of security, particularly for women because of the concerns revealed around differential arrangements for maternity and sickness pay. For women GPs, the time of increased financial pressures from buying into a practice is likely to coincide with plans for childrearing and maternity leave, potentially reducing uptake of partnership roles because of lower earnings or the need to pay locum costs. Although the funding associated with NHS England’s ‘New to Partnership Payment’ (N2PP) scheme may help with this, the findings in the current study show scant awareness of this scheme and concern around the terms of support.^40^ Further use of such schemes may also benefit GPs who described lacking the business skills needed for partnerships.

Women also described first-hand experiences of difficult pay negotiations and fears around risking working relationships. These experiences, together with those described around gender stereotypes from patients and colleagues, suggest that the culture in general practice has room for improvement. Perhaps further support could be offered through the Royal College of General Practice, modelled on the Women in Surgery initiative (see https://www.rcseng.ac.uk/careers-in-surgery/women-in-surgery/).

Larger partnerships may enable more flexibility in roles, with more and greater levels of ancillary staff and opportunities for GPs to diversify and specialise. Previous research with Scottish GPs suggests this may encourage uptake of partnership roles compared with smaller or single-partner practices (Watson V, Schulz R, Murchie P, *et al.* Exploring the business organisation of General Practice partnerships. Unpublished report; 2021). In the current study, some women in the focus groups, however, described feeling less able to progress in larger practices as a result of their voices being lost.

In conclusion, the relative attractiveness of salaried, locum, or private roles in general practice appears to be discouraging both men and women from partnerships because of a range of barriers at individual, organisational, and national levels. Long-standing gendered experiences also remain, affecting the career decisions of women GPs particularly. Promoting positive workplace cultures through strong role models, improved flexibility in roles, and management skills training could encourage greater uptake.

## Data

Materials and data used for the conduct of this research are available from the study authors on request.
